# Sex differences modulate olfactory bulb volume–function relationships but not olfactory training-induced plasticity

**DOI:** 10.1007/s00429-026-03126-4

**Published:** 2026-05-28

**Authors:** Yi-Ting Hsieh, Yin-Chun Liao, Heather Grace Piluden Dulnuan, Jiing-Feng Lirng, Yun-Ting Chao

**Affiliations:** 1 Division of Rhinology, Department of Otorhinolaryngology-Head and Neck Surgery, Taipei Veterans General Hospital, No.201, Sec. 2, Shipai Rd., Beitou District, Taipei, 11217 Taiwan; 2 School of Medicine, College of Medicine, National Yang Ming Chiao Tung University, Taipei, Taiwan; 3 Cagayan Valley Medical Center, Cagayan, Philippines; 4 Department of Radiology, Taipei Veterans General Hospital, Taipei, Taiwan

**Keywords:** Sex differences, Olfactory bulb, Olfactory training, Olfactory bulb plasticity, Olfactory bulb volume

## Abstract

Men typically display larger olfactory bulb (OB) volumes, while women demonstrate superior olfactory performance. Olfactory training (OT) has been shown to induce structural changes in OB, but whether sex influences OT-related plasticity remains unclear. This study aimed to examine how sex differences modulate OB volume–function relationships and OT-related OB neuroplasticity. Participants included 27 women and 11 men with acquired olfactory dysfunction who underwent Sniffin’ Sticks Test and T2-weighted MRI before and after a 12-week OT program of twice-daily exposure to four odorants. OB volume was measured by ITK-SNAP. Pooled baseline analysis revealed no significant correlation between OB volume and composite threshold–discrimination–identification (TDI) score. Women showed a significant correlation between baseline OB volume and TDI on both sides (left: *r* = 0.458, *p* = 0.016; right: *r* = 0.413, *p* = 0.032), whereas no significant associations were observed in men. Following OT, the overall sample showed bilateral OB volume increases (left: mean difference = 4.70 ± 2.16, *p* = 0.036; right: mean difference = 5.64 ± 1.91, *p* = 0.005) and improvements in odor discrimination (t = 2.12, *p* = 0.040) and composite TDI score (t = 2.13, *p* = 0.040). Men consistently presented larger OB volumes (F(1, 36) = 6.63, *p* = 0.014), and no sex-by-session interaction effects were observed. It appears that sex can mask cross-sectional associations between OB volume and olfactory performance, supporting sex-aware analysis when interpreting baseline OB morphometry. In contrast, OT elicits comparable OB volume increases across sexes, suggesting a similar pattern of training-induced structural plasticity.

**Supplementary Information:**

The online version contains supplementary material available at 10.1007/s00429-026-03126-4.

## Introduction

The olfactory bulb (OB) is the first relay linking the peripheral olfactory system to central olfactory pathways in the brain (Yousem et al. 1998). OB volume provides a quantifiable, anatomy-based marker of changes in olfactory loss and recovery; however, several factors must be considered when interpreting OB morphometry. In healthy individuals, OB volume decreases significantly with age (Buschhuter et al. 2008; Kim et al. 2024), whereas no significant correlation is found between age and OB volume in patients with olfactory dysfunction (Hu et al. 2023). Moreover, persistent olfactory loss following an infection has been associated with a more pronounced reduction in OB volume (Yao et al. 2018; Rombaux et al. 2006).

Sex has also been identified as a relevant biological factor influencing olfactory structure and function in both healthy individuals and patients with post-infectious olfactory dysfunction. Although men typically show larger baseline OB volumes (Huart et al. 2019; Hu et al. 2023), women generally exhibit superior olfactory sensitivity (Sorokowski et al. 2019), particularly in response to repeated exposure to benzaldehyde (almond odor) and citralva (lemon odor) (Dalton et al. 2002). Building upon this concept of repeated olfactory stimulation, olfactory training (OT) has been developed as a structured, non-pharmacological intervention for patients with olfactory dysfunction. The classical OT protocol consists of repeated exposure to four odors—rose (phenyl ethyl alcohol), eucalyptus (eucalyptol), lemon (citronellal), and cloves (eugenol)— twice daily for 12 weeks (Hummel et al. 2009). During OT, women also tend to report higher perceived odor intensities (Chao et al. 2022). This discrepancy between structural and behavioral observations complicates the interpretation of sex-related differences in olfactory neurobiology, highlighting the need for further investigation, particularly in the context of therapeutic interventions.

Beyond cross-sectional variation, the OB exhibits longitudinal within-subject changes that are consistent with neuroplasticity (Huart et al. 2013). In the absence of olfactory stimulation, researchers have observed the degeneration of sensory neurons in the olfactory epithelium and the formation of abnormal projections into the OB (Serizawa et al. 2006). Reduced OB volumes have been linked to many olfactory pathologies, including peripheral conditions such as sinonasal inflammation (Rombaux et al. 2008) and post-viral infection (Yao et al. 2018), such as COVID-19–related olfactory dysfunction (Thunell et al. 2022). It has also been linked to central neurodegenerative and psychiatric disorders such as Parkinson’s disease (Wang et al. 2011), Alzheimer’s disease (Thomann et al. 2009), and major depressive disorder (Negoias et al. 2016), as well as mixed etiology such as post-traumatic injury (Jiang et al. 2009). Furthermore, multiple studies have reported a significantly positive correlation between OB volume and olfactory performance in patients with olfactory dysfunction (Baran et al. 2025; Li et al. 2024).

Conversely, increases in OB volume have been reported in parallel with improvements in olfactory function, supporting assertions that the OB is a dynamic structure. For example, patients with chronic rhinosinusitis present significant OB enlargement after endoscopic sinus surgery (Gudziol et al. 2009). Similar volumetric increases have been observed after OT involving repeated exposure to specific odors (Baran et al. 2025; Mahmut et al. 2020). These dynamic changes underscore the highly plastic nature of the OB, attributed to lifelong neurogenesis (Shehata et al. 2018).

This evidence of OB plasticity has prompted the emergence of OT as a non-invasive therapeutic strategy for patients with olfactory dysfunction (Patel 2017). Neuroimaging studies have shown that OT promotes volumetric increases in the OB and olfactory-related brain regions such as hippocampus, thalamus, and cerebellum (Gellrich et al. 2018). It also promotes cortical thickening in the frontal cortex and insula (Rezaeyan et al. 2022), as well as alterations in functional connectivity (Kollndorfer et al. 2015). (Vance et al. 2024) The efficacy of OT has been attributed to neuroplasticity in the olfactory system; however, the precise neurophysiological mechanisms underlying OT have yet to be elucidated. It has been posited that OT augments OB volume by stimulating neurogenesis in glomerular dopaminergic interneurons and the subventricular zone (Marin et al. 2020). Moreover, it has been largely assumed that OT modulates olfactory processing via a bottom-up mechanism involving peripheral stimulation of the olfactory epithelium and its receptors (Hummel et al. 2018). Interestingly, in healthy individuals, unilateral OT has been shown to increase OB volume on both the trained (ipsilateral) and untrained (contralateral) sides, implying that top-down processes also contribute significantly to OB modulation (Negoias et al. 2017).

Despite having established an association between OT and increases in OB volume, relatively few longitudinal studies have examined the factors that influence the neuroplastic response. In particular, it is currently unclear whether sex moderates OT-related changes in OB volume. Moreover, it is uncertain whether structural changes consistently translate into functional improvement, and if so, which components of olfactory function are most affected. In this study, we examined sex-related differences in OB volume and the determinants of OB neuroplasticity following OT.

## Materials and methods

### Subjects

This study included patients who underwent magnetic resonance imaging (MRI) as part of our *multisensory olfactory training program* at our hospital. The inclusion criteria comprised adults (18–80 years) presenting with olfactory dysfunction for a duration exceeding one month. Patients were randomly assigned to one of two different OT modalities. This included a multisensory OT (MOT) group that received conventional odor exposure in conjunction with audiovisual stimulation, and a conventional OT (COT) group underwent odor exposure only.

Patients were excluded if they had congenital anosmia, structural abnormalities of the olfactory cleft or brain, intracranial hemorrhage, history of head trauma, olfactory impairment secondary to craniotomy, sinonasal inflammatory disease with fluctuating symptoms, or psychiatric disorders meeting DSM-IV Axis I or II criteria, including major depressive disorder. Patients were also excluded if MRI quality was inadequate due to motion artifacts or other image distortions.

All participants underwent baseline psychophysical olfactory testing as well as a comprehensive otolaryngological evaluation, including nasal endoscopy, to exclude intranasal pathology. Demographic and clinical data (age, sex, and disease duration) were recorded.

This study was conducted in strict adherence to the Declaration of Helsinki and was approved by the local ethics committee of Taipei Veterans General Hospital (2023-02-006BCF). Written informed consent was obtained from all participants.

### Olfactory training

All eligible participants completed a 12-week OT program. Baseline olfactory testing and MRI were performed before training, and both assessments were repeated at the end of the program. The training protocol required that participants sniff four odorants twice daily for approximately 20 seconds per odorant. The odorants included rose (phenylethanol, #77861), eucalyptus (eucalyptol, #C80601), lemon (citronellal, #27470), and clove (eugenol, #W246700) (all from Sigma-Aldrich, Steinheim, Germany). This procedure was conducted in accordance with the protocol proposed by Hummel et al., which involved applying 1 mL of the undiluted substance onto a cotton pad, which was then stored in a 50 mL amber glass bottle (Hummel et al. 2009).

Participants randomly assigned to the MOT group performed OT while concurrently watching a synchronized 3-minute video clip corresponding to the four odors on a mobile device (Li et al. 2023). Participants assigned to the COT group performed the same OT without concurrent audiovisual stimulation. The patients recorded odor intensity in a smell diary after each session. Compliance was calculated as the proportion of completed records across the 3-month trial.

### Olfactory function assessment

Psychophysical assessment of olfactory function was assessed using the Sniffin’ Sticks Test (Burghart Messtechnik GmbH, Holm, Germany), which consisted of three subtests evaluating the odor threshold, odor discrimination, and odor identification (Oleszkiewicz et al. 2019). Odor thresholds for phenyl ethyl alcohol were determined using a three-alternative, forced-choice, single-staircase procedure. Odor discrimination was evaluated using 16 triplets of odors (two identical, one different). Odor identification was assessed with 16 odors culturally adapted for the Taiwanese population (Shu et al. 2007). The composite threshold–discrimination–identification (TDI) score was calculated as the sum of the three subtests and categorized as normosmia (> 30.5), hyposmia (16.5–30.5), or anosmia (≤ 16.5) (Rumeau et al. 2016).

### Image acquisition

All MRI scans were performed at Taipei Veterans General Hospital using a 3.0 T Philips Ingenia Elition X scanner (Philips Healthcare, Best, The Netherlands) equipped with a 32-channel head coil. High-resolution structural T2-weighted images specifically targeting the OB were acquired in the coronal plane using the following parameters: repetition time (TR) = 3741 ms, echo time (TE) = 100 ms, flip angle = 90°, and an echo train length of 15. Images were obtained with a slice thickness of 2 mm without inter-slice gap and reconstructed to a matrix of 512 × 512.

### Olfactory bulb volume measurement

OB volume was measured via manual segmentation using ITK-SNAP software (version 4.2.2, www.itksnap.org) (Yushkevich et al. 2006) in accordance with an established protocol (Joshi et al. 2020). To ensure consistency across raters, this study applied standard ITK-SNAP settings, including a 4× zoom level, paintbrush size of 2, and overall opacity of 25%. After identifying slices with clear visualization of the OB, segmentation began at the most anterior slice. Each OB was manually delineated using smooth, outwardly extending contours, proceeding posteriorly through consecutive slices to the posterior boundary, defined by an abrupt reduction in OB diameter at the transition to the olfactory tract. Volumes of the right and left OBs were independently calculated by two raters (Liao YC and Dulnuan HP) blinded to all diagnostic and clinical data. In cases where inter-rater differences exceeded 10%, the corresponding author (Chao YT) repeated the measurement, whereupon the final OB volume was calculated as the mean of the two closest values that fell within the 10% threshold.

### Statistical analyses

Independent t-tests were used to compare continuous variables between groups. Paired t tests were used to evaluate pre- to post-training changes in OB volume and in olfactory outcomes, including threshold, discrimination, identification, and the composite TDI score. Repeated-measures ANOVA was performed to evaluate the main effects and interactions influencing OB volume changes. In this model, session (pre- vs. post-OT) and side (left vs. right OB) were defined as within-subject variables, whereas sex (men vs. women), age (≤ 45 vs. >45 years old, median age), baseline olfactory status (anosmia vs. hyposmia), duration of dysfunction (< 1 vs. ≥1 year), OT modality (COT vs. MOT), and responsiveness (TDI improvement ≥ 5.5 vs. <5.5, minimal clinical important difference, MCID) (Gudziol et al. 2006) were included as between-subject factors. Pearson’s correlation analysis was used to assess the relationship between olfactory function and OB volumes. A p-value of < 0.05 was considered statistically significant. All statistical analysis was performed using IBM SPSS Statistics for Windows, version 28 (IBM Corp., Armonk, N.Y., USA).

## Results

### Patient demographic data

Table 1 lists the demographic characteristics of the 38 patients included in this study. The mean age was 44.4 ± 13.2 years (range: 22–67 years). Based on Sniffin’ Sticks test results, all patients were diagnosed with hyposmia or anosmia. Baseline OB volumes were as follows: left side (58.00 ± 19.72 mm³) and right side (59.66 ± 22.00 mm³). OB volumes were larger in men than women (left side: mean difference = 16.88 ± 6.57, t = 2.57, *p* = 0.015; right side: mean difference = 18.12 ± 7.39, t = 2.45, *p* = 0.019), and TDI scores were higher in women than men (composite TDI: t = −2.26, *p* = 0.030; threshold: t = −2.21, *p* = 0.034, discrimination: t = −2.05, *p* = 0.048), despite no significant sex differences in terms of age, disease duration, training compliance, or etiology. 

The MOT and COT groups included 21 and 17 participants, respectively. The two groups were comparable in age (mean age: MOT = 46.05 ± 13.61 years; COT = 42.35 ± 12.70 years; t = 0.857, *p* = 0.397). The etiologies of olfactory dysfunction were also similar between groups (postviral: MOT = 12, COT = 15; idiopathic: MOT = 9, COT = 2; Fisher’s exact test, *p* = 0.070).

**Table 1:** Demographic data

Variables	Total	Men	Women	t	*p* value
Subjects, *N*	38	11 (29%)	27 (71%)		
Age, years	44.4 ± 13.2	43.6 ± 11.5	44.7 ± 14.0	−0.25	0.804
Disease duration, weeks	90.5 ± 139.8	96.2 ± 151.1	88.2 ± 137.8	0.16	0.875
Baseline OB volume					
Left, mm^3^	58.00 ± 19.72	69.99 ± 20.42	53.11 ± 17.53	2.57	0.015*
Right, mm^3^	59.66 ± 22.00	72.54 ± 25.12	54.42 ± 18.65	2.45	0.019*
Baseline TDI score					
Composite TDI	19.51 ± 6.86	15.77 ± 6.42	21.03 ± 6.54	−2.26	0.030*
Threshold	1.95 ± 1.36	1.41 ± 0.63	2.18 ± 1.52	−2.21	0.034*
Discrimination	8.61 ± 3.21	7.00 ± 3.23	9.26 ± 3.02	−2.05	0.048*
Identification	8.96 ± 3.64	7.36 ± 3.50	9.59 ± 3.56	−1.76	0.087
Baseline status					
Anosmia	15	7	8		^#^0.073
Hyposmia	23	4	19		
Etiology					
Postviral	27	6	21		^#^0.238
Idiopathic	11	5	6		
OT modality, *N*					
MOT	21	8	13		^#^0.282
COT	17	3	14		
Compliance, %	89.34 ± 14.24	85.73 ± 14.38	90.81 ± 14.19	−1.00	0.325

Continuous variables: mean ± standard deviation; categorical variables: N (%)

*OB* Olfactory bulb, *OT* Olfactory training, *MOT* Multisensory olfactory training, *COT* Conventional olfactory training

*p < 0.05

^#^Analyzed using Fisher’s exact test

### Correlation between baseline OB volume and olfactory function

In the overall cohort, no significant correlations were observed between baseline TDI scores and baseline OB volumes on either side. However, men had significantly larger baseline OB volumes than women, despite significantly lower baseline TDI scores (Table 1). Given these sex differences, correlation analyses were conducted separately for men and women.

**Fig. 1:**
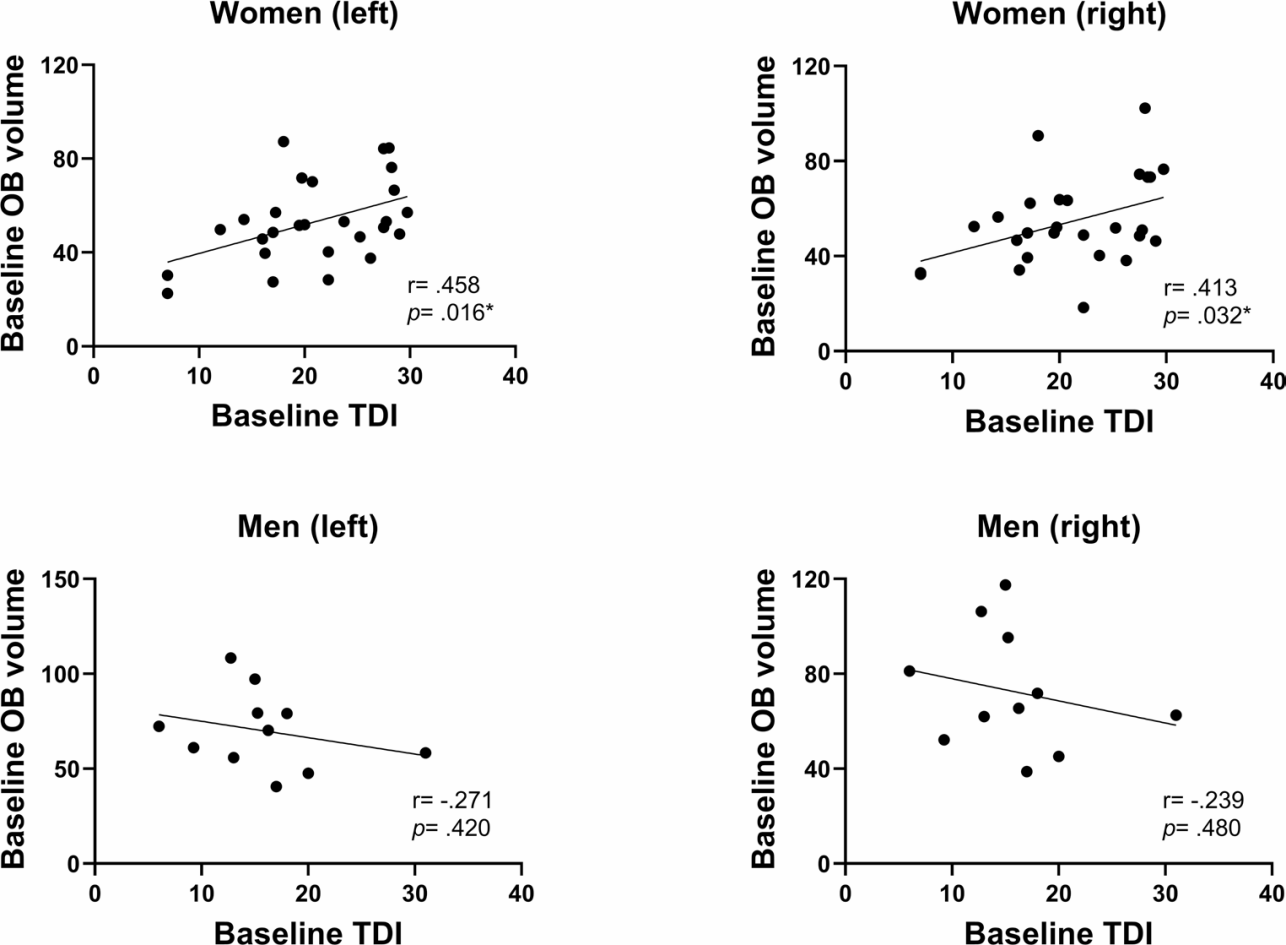
Correlations of baseline OB volume and baseline olfactory function by sex. *p < 0.05

Among women, right baseline OB volume was positively correlated with odor discrimination (*r* = 0.485, *p* = 0.010) and composite TDI score (*r* = 0.413, *p* = 0.032). Left OB volume showed similar associations with odor discrimination (*r* = 0.539, *p* = 0.004) and TDI score (*r* = 0.458, *p* = 0.016). No significant correlations were observed in men (*p* > 0.05) (Fig. 1).

### Changes in OB volumes and olfactory function following OT

**Fig. 2:**
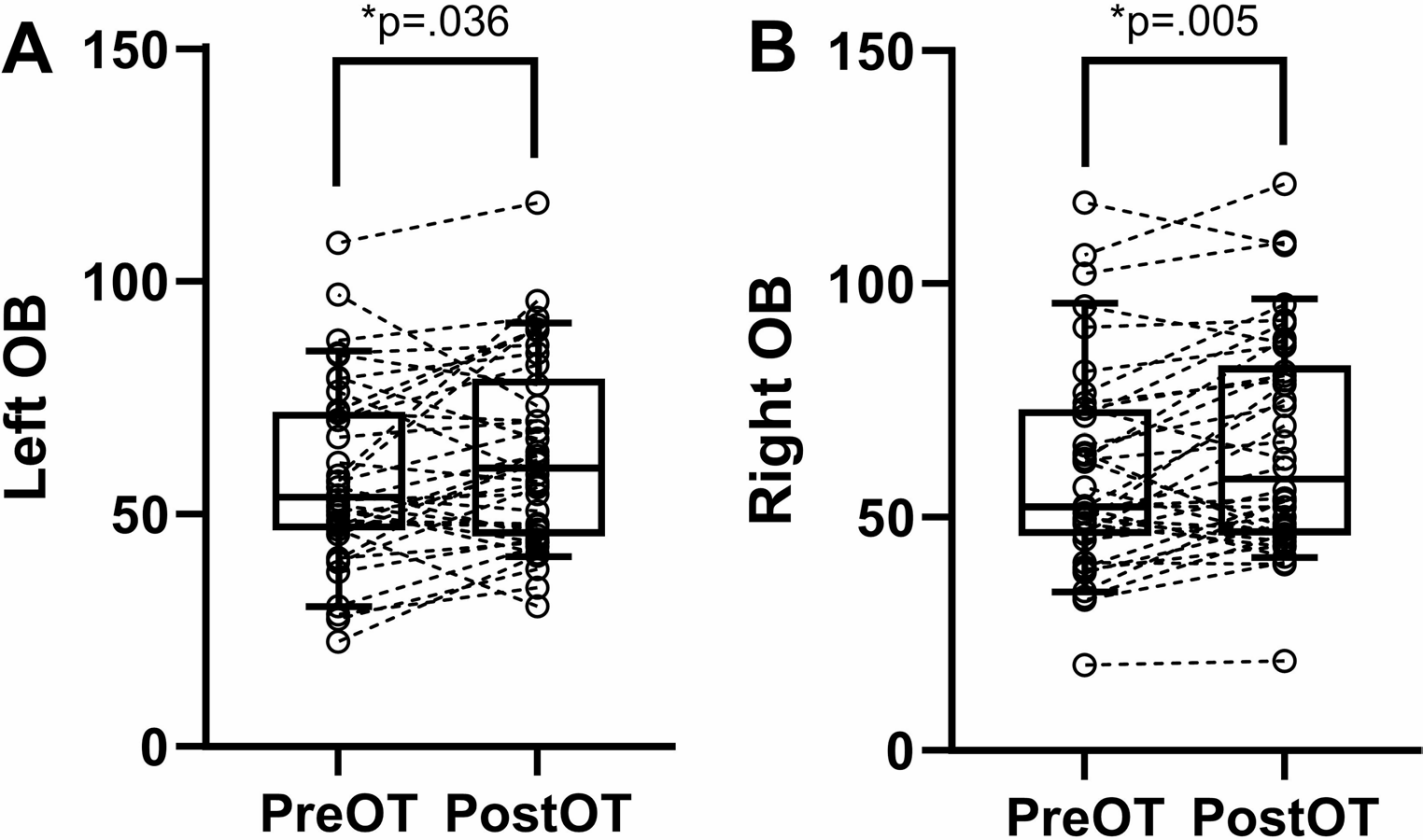
Changes in OB volume between pre-OT and post-OT. *p < 0.05

**Fig. 3:**
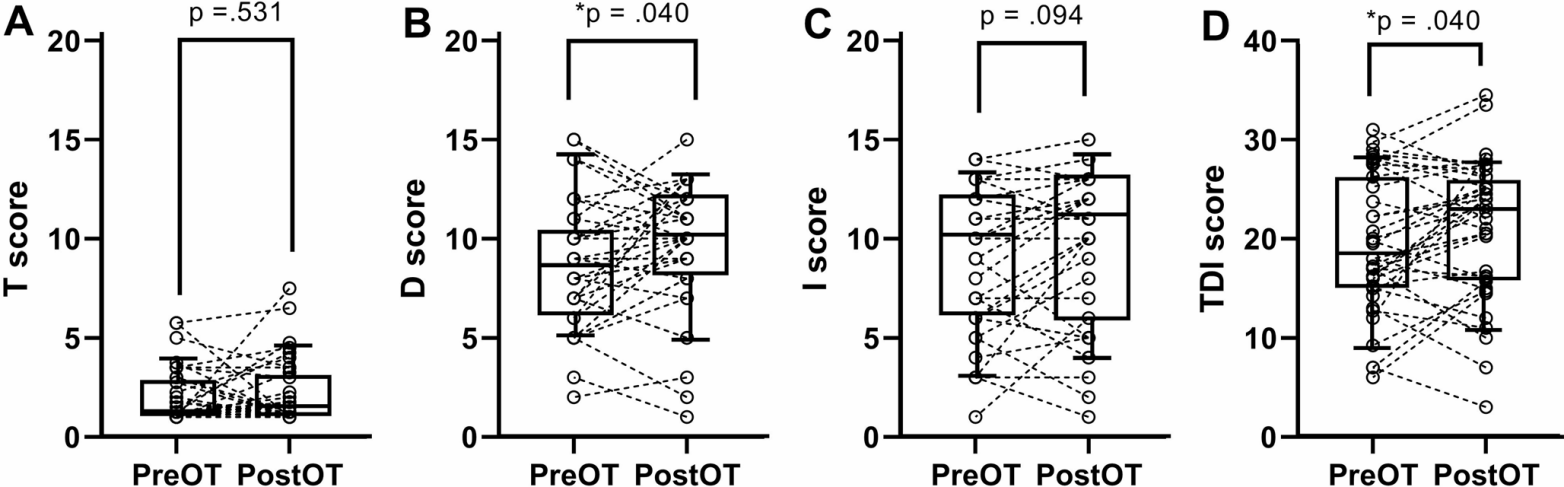
Changes in odor threshold (T), discrimination (D), identification (I), and the composite TDI score between pre-OT and post-OT. *p < 0.05

Following the completion of OT, the mean OB volumes were as follows: left side (62.69 ± 20.26 mm³) and right side (65.31 ± 23.24 mm³). Significant increases in OB volume were observed on both sides (left: mean difference = 4.70 ± 2.16, t = 2.20, *p* = 0.036; right: mean difference = 5.64 ± 1.91, t = 2.95, *p* = 0.005) (Fig. 2). Significant improvements were observed in discrimination (t = 2.12, *p* = 0.040) and composite TDI score (t = 2.13, *p* = 0.040). No significant changes were observed in odor identification (t = 1.72, *p* = 0.094) or threshold (t = 0.632, *p* = 0.531) (Fig. 3).

### Factors associated with changes in OB volume

**Fig. 4:**
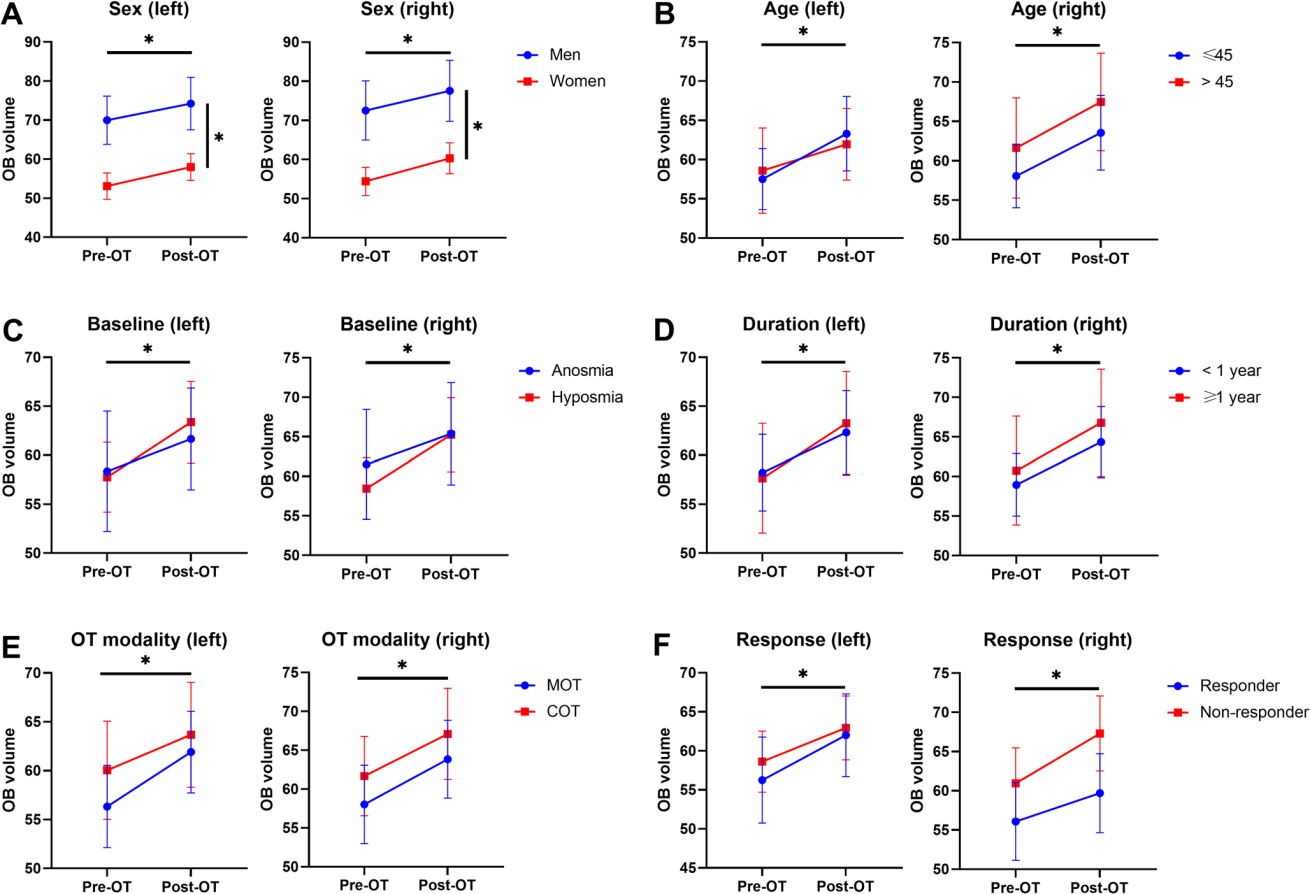
Changes in olfactory bulb volume after olfactory training across subgroups. *p < 0.05

Repeated-measures ANOVA revealed a significant main effect of session. This main effect remained significant when evaluating sex, age, baseline olfactory status, duration of dysfunction, OT modality, and responsiveness as between-subject factors (Sex: F(1, 36) = 5.486, *p* = 0.025; Age: F(1, 36) = 6.872, *p* = 0.013; Baseline olfaction: F(1, 36) = 6.142, *p* = 0.018; Duration: F(1, 36) = 7.085, *p* = 0.012; OT modality: F(1, 36) = 6.857, *p* = 0.013; Response: F(1, 36) = 5.171, *p* = 0.029). These results indicate an overall increase in OB volume between pre-OT and post-OT on both sides across subgroups. A significant main effect of sex was also observed (F(1, 36) = 6.63, *p* = 0.014), indicating that across sessions, OB volumes were larger among men than among women (Fig. 4A). No significant interaction effects were detected between session and between-subject factors. No significant main or interaction effects involving side (left vs. right) were observed (Fig. 4).

To address the potential influence of baseline olfactory function between sexes, additional repeated-measures ANCOVA analyses controlling for baseline TDI scores as a covariate were performed. Baseline TDI did not significantly influence changes in OB volume (F(1, 35) = 2.11, *p* = 0.155), and the main effect of sex remained significant after adjustment (F(1, 35) = 8.76, *p* = 0.006). The interaction effects between session and sex also remained non-significant (F(1, 35) < 0.01, *p* = 0.957).

## Discussion

In the current study, baseline OB volumes were significantly larger in men than in women, whereas baseline TDI scores were significantly lower in men. Previous MRI studies have similarly reported that OB volumes are larger in adult men than in adult women (Buschhuter et al. 2008). Taken together, this pattern suggests that sex-based comparisons deviate from the conventional assumption that larger OB volumes are associated with superior olfactory performance. One explanation is that sex differences in OB volume partly reflect overall differences in brain size, as men typically have larger brain volumes (Rombaux et al. 2009). Supporting this assertion, one previous study reported a strong correlation between intracranial volume and regional brain volumes, including the cortex, white matter, and thalamus, and found that intracranial volume was more informative than sex for explaining inter-individual variation in regional volumes (Liu et al. 2025). To further address this issue, we performed supplementary analyses normalizing OB volume to intracranial volume (see Supplement file 1). These analyses suggest that although sex differences in OB volume are partly attributable to intracranial volume, the sex-dependent relationship between OB volume and olfactory function cannot be fully explained by intracranial volume alone, suggesting that additional sex-specific structural–functional mechanisms may underlie this relationship.

To better understand the structural basis of sex dimorphism, OB cellularity has been investigated as a measure that may relate more directly to olfactory function. It has been reported that compared to men, women have more neuronal cells and higher neuronal cell density in the OB (Oliveira-Pinto et al. 2014). Consistent with the possibility of more extensive central processing, functional imaging studies have also shown that when exposed to the same odorants, women exhibit a larger extent of activation in frontal and perisylvian regions (Yousem et al. 1999). Together, these findings support the view that olfactory information is processed more extensively in women at the level of OB microstructure and cortical engagement.

A growing body of behavioral data indicates that women significantly outperform men across most aspects of olfactory function (Sorokowski et al. 2019). Several mechanisms have been proposed to explain this. Endocrine factors, such as estrogen, were shown to be associated with increases in threshold-level olfactory sensitivity (Schneider et al. 1958); progesterone was also reported to modulate olfactory neural network during the periovulatory period of the menstrual cycle (Chao et al. 2023). Sociocultural factors may also play a role, as women tend to exhibit greater odor awareness from early childhood and traditionally engage more frequently in odor-related activities such as food preparation (Nováková et al. 2014). Accordingly, sex differences in olfactory function likely reflect a combination of hormonal modulation and experience-dependent influences, in addition to structural differences.

Researchers have reported a positive correlation between OB volume and olfactory function. Considering the evidence for sex differences and the fact that no significant correlation was observed in our pooled analysis, we examined this relationship separately in men and women. Following stratification, we observed a significantly positive correlation between OB volume and olfactory function in women, whereas no significant association was detected in men. The negative trend in men should be interpreted with caution and may reflect limited statistical power due to the relatively small sample size. Another potential explanation is that the proportion of anosmic individuals tended to be higher among men. Because the Sniffin’ Sticks test employs a forced-choice paradigm, anosmic patients may achieve correct responses by chance, which limits the interpretability of their scores (Whitcroft et al. 2017; Chao et al. 2025). Conversely, the female cohort had a higher proportion of hyposmic individuals, resulting in a more reliable correlation in this group. Taken together, these findings suggest that sex differences obscure the association between OB volume and olfactory performance when both sexes are analyzed together. This underscores the importance of considering sex as a biological variable when interpreting OB volumes.

This insight is a key contribution of the study. We recommend that future investigations of cross-sectional correlation between OB volume and olfactory function account for sex in both measurement and statistical analysis to ensure that sex-related effects are appropriately characterized and reduce the risk of misinterpretation. We also suggest that prior studies reporting no association between OB size and olfactory function be reconsidered, given the possibility that pooling men and women may mask sex-specific relationships. While single-sex cohorts may reduce variability, particularly in hypothesis-driven studies, our findings support sex-stratified analyses in cross-sectional studies and do not support sex-specific recruitment in longitudinal settings.

Throughout the training session, the OB volumes in men were larger than those in women. Importantly, no interaction effect was observed between session and sex, suggesting that the magnitude of the volume increase was similar in both sexes. A previous study of lateralized OT also reported no significant sex effect on OB enlargement on either side (Negoias et al. 2017). Considering that OT provides the same degree of olfactory stimulation to all participants, it is plausible that both sexes exhibit a similar neuroplastic response despite baseline OB differences. Thus, our findings provide novel evidence that OT-induced OB neuroplasticity is comparably modulated in men and women.

This result further suggests that, in longitudinal designs such as pre- and post-OT comparisons, sex stratification may be less critical when the primary outcome is within-subject change in OB volume. From a clinical perspective, the comparable training-related OB neuroplasticity observed in men and women supports OT as a rehabilitation approach for both sexes.

The observed increase in OB volume after OT indicates that repeated exposure to odorants is associated with structural neuroplasticity. Prior work has similarly shown that increasing peripheral olfactory input can promote OB plasticity. In animal models, restoration of odor stimulation has been reported to reverse OB volume reductions that occur after early reversible olfactory deprivation (Cummings et al. 1997). OB recovery and functional gains have also been achieved using clinical interventions aimed at restoring peripheral inputs, such as functional endoscopic sinus surgery for chronic rhinosinusitis patients (Gudziol et al. 2009) and olfactory rehabilitation aimed at restoring orthonasal air flow for laryngectomy patients (Gurbuz et al. 2022). Even in healthy individuals, continuous olfactory exposure (e.g., sommelier training) has been linked to OB enlargement (Filiz et al. 2022). These studies suggest that OB plasticity can be promoted by treatments or experiences that enhance peripheral sensory input.

In the present study, improvements in olfactory function were primarily observed in odor discrimination, with a non-significant trend toward improvement in odor identification. Given that odor discrimination relies on higher-order perceptual and cognitive processing, these findings suggest that OT may preferentially engage top-down mechanisms. This pattern is consistent with prior neuroimaging findings showing that OT is associated with changes in functional connectivity and with volume increases in olfactory-related brain regions (Gellrich et al. 2018; Rezaeyan et al. 2022; Kollndorfer et al. 2015). It is also supported by the observation of bilateral OB enlargement following unilateral training (Negoias et al. 2017).

Conversely, researchers have suggested a bottom-up mechanism based on a significant correlation between changes in OB volume and odor threshold (a peripheral function) in patients with chronic olfactory loss (Gudziol et al. 2009; Haehner et al. 2008). Furthermore, OT can induce peripheral neuronal plasticity, leading to the restoration of neuroepithelial activity (Chen et al. 2025). Thus, it appears that the outcomes of OT-induced OB plasticity are mediated by a complex interplay of both top-down and bottom-up mechanisms.

Although significant OB enlargement was observed after OT, only 23% of participants achieved the MCID in the composite TDI score. This discordance suggests that OB volume may be more sensitive than psychophysical measures for detecting training-related changes. This interpretation is consistent with assertions in previous studies that OB volume may capture subtle variations in olfactory function that are not necessarily reflected in psychophysical testing (Rombaux et al. 2008).

In the present study, participants underwent either MOT or COT. Although these protocols differ in modality, they share identical odorants, training frequency, and duration. The primary distinction lies in the addition of audiovisual cues in the MOT protocol, which are intended to engage higher-order sensory and associative processes while preserving the same olfactory stimulation. From a mechanistic perspective, both training paradigms provide equivalent olfactory input, which is considered the principal driver of OB plasticity. Accordingly, any potential influence of the additional audiovisual components would be expected to occur at higher-order processing levels rather than at the level of the OB. Consistent with this framework, our results showed no significant differences between MOT and COT in OB volume, and no interaction between training modality and session. These findings suggest that the type of OT protocol did not significantly influence the observed outcomes. Therefore, the two training groups were combined in the main analyses to increase statistical power.

### Limitations

This study has several limitations that should be considered in the interpretation of our findings. First, a relatively small sample size and unbalanced sex distribution limited statistical power and generalizability. This issue is common in longitudinal neuroimaging studies requiring paired pre- and post-training MRI scans. However, all participants were scanned using the same MRI machine, minimizing imaging variability. Second, we did not divide participants into subgroups based on the duration of olfactory dysfunction. In our sample, duration was not significantly related to baseline OB volume or changes in OB volume after training. This suggests that differences in duration were unlikely to have materially influenced the main findings. Third, the age range was wide. Nonetheless, the mean age did not differ between men and women, reducing the likelihood that age confounded the observed sex-related effects.

## Conclusions

When the cohort was analyzed as a whole, baseline OB volume was not significantly associated with baseline olfactory performance. In contrast, sex-stratified analyses identified a significant positive association between OB volume and the composite TDI score in women. This pattern suggests that sex-related differences may obscure cross-sectional volume–function relationships when men and women are analyzed together. This finding supports treating sex as a biological variable in studies that interpret OB volume in relation to olfactory function. It also suggests that null findings in pooled analyses do not necessarily exclude meaningful sex-specific associations.

OT was associated with a significant increase in OB volume in both sexes. Importantly, the absence of a session-by-sex interaction suggests that the magnitude of OT-related OB enlargement was comparable in men and women, despite baseline differences in OB volume. The observed improvements in odor discrimination is consistent with training effects on higher-order olfactory processing. These findings suggest that OT may engage top-down modulation in addition to peripheral-driven structural plasticity. 

Our findings underline the need for future studies with larger cohorts, a balanced sex representation, and analytic approaches that explicitly test sex-specific associations to clarify the mechanisms underlying sex-related differences and determine how these differences relate to the OB volume–function relationship, training-induced plasticity, and clinical outcomes.

## Supplementary Information

Below is the link to the electronic supplementary material.

Supplementary Material 1

## Data Availability

The datasets generated and/or analyzed during this study are not publicly available to protect individual privacy. However, they can be obtained from the corresponding author upon reasonable request.
