# Supplementary material for: Sex differences modulate olfactory bulb volume–function relationships but not olfactory training-induced plasticity
Source: Brain Struct Funct. 2026 May 28;231(5):73. doi: 10.1007/s00429-026-03126-4 (PMC13219168; doi:10.1007/s00429-026-03126-4)
Supplement: Supplementary file 1 — Supplementary Material 1 [file 429_2026_3126_MOESM1_ESM.docx]

Supplementary file

**Intracranial Volume Estimation and Its Effect on Olfactory Bulb Volume**

**Methods**

High-resolution structural T1-weighted images were acquired on a 3T Philips Ingenia Elition X scanner. The acquisition parameters were: repetition time (TR) = 7.9 ms, echo time (TE) = 2.719 ms, flip angle = 8°, and 184 sagittal slices with a 1 mm slice thickness. The acquisition matrix was 250 × 250, which was further reconstructed to 512 × 512, resulting in an isotropic voxel size of 1 × 1 × 1 mm³.

T1-weighted structural images were segmented into gray matter (GM), white matter (WM), and cerebrospinal fluid (CSF) using the unified segmentation procedure implemented in SPM12 (Statistical Parametric Mapping; Wellcome Trust Centre for Neuroimaging, University College London). Tissue probability maps (c1–c3) were generated for each subject. Total intracranial volume (TIV) was calculated as the sum of GM, WM, and CSF volumes (cm^3^).

**Results**

To examine the potential influence of global brain size, we first evaluated the relationship between TIV and olfactory bulb (OB) volume. The TIV showed significant positive correlations with both right (r = .353, p = .030) and left (r = .341, p = .036) OB volumes in the overall cohort, indicating that OB size partly scales with global brain volume.

We next examined OB volume normalized to TIV (OB/TIV). While men exhibited significantly larger baseline OB volumes than women in the primary analysis, no significant sex difference was observed in normalized OB volume (right: t = 1.46, p = .154; left: t = 1.44, p = .157).

We then evaluated the relationship between normalized OB volume and olfactory performance. Similar to the primary analysis, normalized OB volume showed no significant correlations with baseline T, D, I, or composite TDI scores when men and women were analyzed together. Sex-stratified analyses showed a pattern comparable to the main results, with significant associations between OB volume and olfactory performance observed in women but not in men.

When normalized OB volume was used as the outcome variable in the repeated-measures ANOVA, a significant main effect of session remained, indicating a training-related increase in OB volume. However, the previously observed sex difference was no longer statistically significant.

Taken together, these analyses suggest that while baseline sex differences in absolute OB volume are partly attributable to global brain size scaling, the sex-dependent relationship between OB volume and olfactory function cannot be fully explained by differences in intracranial volume.
